# The Role of DPP4 Activity in Cardiovascular Districts: In Vivo and In Vitro Evidence

**DOI:** 10.1155/2013/590456

**Published:** 2013-06-18

**Authors:** L. Pala, C. M. Rotella

**Affiliations:** Section of Endocrinology, Department of Biomedical, Experimental and Clinical Sciences, University of Florence, Viale Pieraccini 6, 50134 Florence, Italy

## Abstract

The introduction of incretin hormone-based therapies represents a novel therapeutic strategy, since these drugs not only improve glycemia with minimal risk of hypoglycemia, but also have other extraglycemic beneficial effects. These agents, which are effective in improving glucose control, could also have positive effects on the incidence of cardiovascular events. The aim of this review is to summarize the present literature about the role of dipeptidyl peptidase 4 (DPP4) in cardiovascular districts, not only strictly correlated to its effect on glucagon-like peptide-1 (GLP-1) circulating levels, but also to what is known about possible cardiovascular actions. Actually, DPP4 is known to be present in many cells and tissues and its effects go beyond purely metabolic aspects. Almost always the inhibition of DPP4 activity is associated with improved cardiovascular profile, but it has shown to possess antithrombotic properties and these different effects could be connected with a site and/or species specificity of DPP4. Certainly, DPP4 seems to exert many functions, both directly and indirectly, on cardiovascular districts, opening new possibilities of prevention and treatment of complications at this level, not only in patients affected by diabetes mellitus.

## 1. Introduction 

Glucagon-like peptide-1 (GLP-1) receptor agonists and dipeptidyl peptidase 4 (DPP4) inhibitors are currently used as glucose-lowering agents in type 2 diabetes, due to their effects on insulin and glucagon secretion. Many reviews show that these agents, which are effective in improving glucose control, could also have a beneficial effect on the incidence of cardiovascular (CV) events. The analysis of major CV events reported during trials with metabolic endpoints shows a significant reduction of risk with both classes of drugs. Longer-term trials specifically designed to assess the effects of GLP-1 receptor agonists and DPP4 inhibitors on major cardiovascular events are currently ongoing. Available data suggest that incretin-based therapies could prevent cardiovascular disease via multiple mechanisms; in particular, pilot studies in humans show that GLP-1 receptor agonists and DPP4 inhibitors are capable of ameliorating myocardial function and protect myocardiocytes from ischemic damage, independent of their glucose-lowering effects, and both classes of drugs enhance endothelial function [1–3]. In clinical studies, both GLP-1 receptor agonists and DPP4 inhibitors improve *β*-cell function indexes; besides all these agents show trophic effects on beta-cell mass in animal studies. Their use is associated with positive or neutral effect on body weight and improvements in blood pressure, diabetes-associated dyslipidemia, hepatic steatosis markers, and myocardial function [4, 5]. The sum of such effects potentially reduces the burden of cardiovascular disease, which is a major cause of mortality in patients affected by diabetes mellitus [6]. These hypotheses advanced from evidence in animal model studies, and from multiple meta-analyses and a retrospective study in humans. However, no randomized controlled trials with this primary end point have been published to date. But which is the role of DPP4 on cardiovascular system? The aim of the present review is to summarize the present literature about the role of DPP4 in cardiovascular districts not only correlated to its inhibition, but also to what is known about its possible CV effects. A polymorphism in the DPP4 gene in patients with known CAD may increase the risk of MI. Such polymorphism is associated with decreased plasma DPP4 level in patients with MI [7]. DPP4 is expressed on the surface of several cell types [8], being a transmembrane glycoprotein, with serine exopeptidase activity that cleaves X-proline dipeptides from the N-terminus of polypeptides. Protein dimerization is required for catalytic activity, and glycosylation of the enzyme impacts its physiological functions. DPP4 substrates are proline- or alanine-containing peptides, which include growth factors, chemokines, neuropeptides, and vasoactive peptides [9]. Therefore, DPP4 is not specific for GLP-1 and a wide range of DPP4 substrates has been described. Some of these may be cleaved by DPP4 only at very high concentrations especially in supraphysiological conditions. We know that chronic hyperglycaemia induces a significant increase in DPP4 activity in type 1 and type 2 diabetes. This phenomenon could contribute to the reduction in circulating active glucagon-like peptide-1 and to the consequent postprandial hyperglycaemia in type 2 diabetic patients with poor metabolic control [10]. DPP4 is considered a novel adipokine that may impair insulin sensitivity in an autocrine and paracrine fashion. Furthermore, DPP4 release strongly correlates with adipocyte size, potentially representing an important source of DPP4 in obesity. Therefore, DPP4 could be involved in linking adipose tissue with the metabolic syndrome [11]. A recent review summarizes very well data on cardiovascular effects of DPP4 inhibitors, with a special interest in GLP-1-independent mechanisms [12]. In the present review we have divided our analysis into three parts: clinical studies, animal model studies, and in vitro evidence, including only those that are correlated with the possible role of DPP4.

## 2. In Vivo Evidence

### 2.1. Human Studies

Several prospective randomized controlled clinical trials in humans with established atherosclerosis have tested the effects of DPP4 inhibition on severe cardiovascular events. In addition to their glucose-lowering and weight-neutral or weight-reducing actions, incretin-based therapies decrease systolic blood pressure and improve fasting and postprandial lipid parameters, by reducing total-cholesterol, low-density lipoprotein-cholesterol, and triglycerides concentrations, increasing also high-density lipoprotein-cholesterol values. Reduced high-sensitivity C-reactive protein levels and improved endothelial dysfunction have been also reported. Incretin-based therapies have several beneficial effects on cardiovascular risk factors and, for this reason, the extension of the use of these drugs in diabetic patients with cardiovascular complications has been recently suggested. Yet, the long-term effects of incretin-based therapies on subclinical or clinical atherosclerosis remain to be established by future studies [13]. DPP4 is known to exert its effects via both enzymatic and nonenzymatic mechanisms. A soluble form of DPP4 lacking the cytoplasmic and transmembrane domain has also been recently recognized. Besides enzymatic inactivation of incretins, DPP4 also mediates degradation of many chemokines and neuropeptides. The nonenzymatic function of DPP4 plays a critical role in providing costimulatory signals to T cells via adenosine deaminase (ADA). DPP4 may also regulate inflammatory responses in innate immune cells such as monocytes and dendritic cells. The multiplicity of functions and targets suggests that DPP4 may play a distinct role aside from its effects on the incretin axis. Indeed recent studies in experimental models of atherosclerosis provide evidence for a robust effect for these drugs in attenuating inflammation and plaque development [14]. In a recent study on healthy young people DPP4 activity is correlated with the clinical parameters of obesity and/or diabetes. DPP4 activity displayed highly significant positive correlation with BMI and a negative significant correlation with the plasma adiponectin concentration (*P* = 0.013), but not with fasting blood glucose [15].

### 2.2. Animal Models

Plasma DPP4 activity was associated with the development of obesity, type 2 diabetes, and type 1 diabetes using animal models; in particular streptozotocin (STZ) is commonly used to induce type 1 and late-phase type 2 diabetic models by selective *β*-cell destruction in small rodents. DPP4 deficiency delays the onset of diabetes but worsens dyslipidaemia and renal dysfunction induced by STZ [16].

Comparing the cardiovascular responses of ischemia/reperfusion between wild-type and DPP4-deficient rats after 45 min of coronary artery occlusion, followed by reperfusion for 2 h, DPP4-deficient rats had better cardiac performance in association with low infarct size and cardiac injury markers (LDH, ANP, and BNP), which could be attenuated, but not completely abrogated, by exendin (9–39), a GLP-1 receptor antagonist, involving a GLP-1 receptor-independent pathway. The benefit of cardiac protective action against ischemia/reperfusion injury was demonstrated in DPP4-deficient rats and it was mediated through both GLP-1 receptor-dependent and receptor-independent mechanisms [17]. The effect of chemical inhibition or genetic deletion of DPP4 activity was studied on the cardiovascular function in normoglycemic and diabetic mice after experimental myocardial infarction. Cardiac structure and function were assessed by hemodynamic monitoring and echocardiography in DPP4 knockout mice versus wild-type littermate controls and after left anterior descending coronary artery ligation-induced myocardial infarction. Increased cardiac expression of phosphorylated AKT (pAKT), pGSK3beta, and atrial natriuretic peptide (ANP) was detected in the nonischemic Dpp4(−/−) heart. Sitagliptin and metformin treatment of wild-type diabetic mice reduced mortality after myocardial infarction. Sitagliptin improved functional recovery after I/R injury ex vivo in WT mice with similar protection from I/R injury also manifest in hearts from Dpp4(−/−) versus Dpp4(+/+) mice [18]. Peripheral artery disease is a potentially incapacitating disease whose pharmacological options are limited. Stromal cell-derived factor-1 (SDF-1) is a chemokine that attracts endothelial progenitor cells and promotes angiogenesis. Therapeutic use of SDF-1 in hindlimb ischemia may be challenged by proteolytic degradation. SDF-1 is a substate of DPP4. SDF-1 engineered to be resistant to DPP4 and matrix metalloproteinase-2 cleavage, and delivered by nanofibers, improves blood flow in a model of peripheral artery disease [19]. In vitro and in vivo, parathyroid hormone (PTH) inhibited the activity of DPP4, which cleaves and inactivates SDF-1. Recent data suggest that PTH inhibits DPP4, leading to an increased concentration of plasma SDF-1*α* and favoring the homing of bone-marrow-derived CXCR4+ EPCs to sites of experimental myocardial infarction in mice [20]. Cleavage of chemokines such as CXCL11, SDF-1, and eotaxin by DPP4 reduces the ability of these proteins to serve as chemoattractants to T cells and monocytes [13].

The inhibition of DPP4 protects the heart from acute myocardial ischemia. However, the role of DPP4 in chronic heart failure, independent of coronary artery disease, remains unclear. Diabetic rats exhibited diastolic left ventricular dysfunction (DHF) with enhanced interstitial fibrosis caused partly by the increased ratio of matrix metalloproteinase-2 to tissue inhibitor of metalloproteinase-2 in a DPP4-dependent fashion. DPP4 inhibition reverses diastolic left ventricular dysfunction viamembrane-bound DPP4/stromal cell-derived factor-1*α*-dependent local actions on angiogenesis and circulating DPP4/glucagon-like peptide-1-mediated inotropic actions. Myocardium-derived DPP4 activity in coronary sinus can be monitored by peripheral vein sampling, which partly correlates with diastolic left ventricular dysfunction index; thus, circulating DPP4 may potentially serve as a biomarker for monitoring diastolic left ventricular dysfunction [21]. In congestive heart failure (HF), plasma B-type natriuretic peptide (BNP) seems devoid of biological effectiveness. BNP(1–32) could be truncated into BNP(3–32) by dipeptidyl peptidase 4 (DPP4), and BNP(3–32) has reduced biological activities. Increased DPP4 activity is associated with pathophysiology of HF. DPP4 activity was measured in dogs with different degree of heart failure (HF): DPP4 activity increased linearly with body weight and was significantly higher in heart failure class 1 compared with healthy heart and heart failure class 3 demonstrating that DPP4 activity could be involved in early stages of heart failure [22].

## 3. In Vitro Evidence

DPP4 is an enzyme produced by endothelial cells in different districts and circulates in plasma. DPP4 activity and mRNA expression were measured in cultured human aortic endothelial cells (HAEC) and human microvascular dermal endothelial cells (HMVEC) exposed to high glucose, metformin, and rosiglitazone. The modulation of DPP4 activity in endothelial cells is site specific; in fact hyperglycemia is able to increase in a significant manner the DPP4 activity only in microvascular endothelial cells [23]. Other authors have confirmed that endothelial effects of GLP-1 are mediated by an increased nitric oxide bioavailability through GLP-1 receptor-dependent and -independent pathways [24]. Recently, in vitro treatment with GLP-1 was also able to increase proliferation of the vasculoprotective endothelial progenitor cells as shown through an action on VEGF [25]. Besides, the relationship between DPP4 activity and angiogenesis may be mediated by modulation of the Neuropeptide Y (NPY) signaling that after being processed by DPP4, NPY1–36 is converted to its shorter form (NPY3–36), thus shifting its activity from Y1-mediated vasoconstriction and vascular smooth muscle cell growth to Y2/Y5-mediated angiogenesis [26]. DPP4 may proteolytically inactivate local mediators involved in gliomagenesis [27].

Almost always the inhibition of DPP4 activity is associated with improved cardiovascular profile; in a recent article it was shown that DPP4 also possesses antithrombotic properties and may behave as an immobilized anticoagulant on endothelial cells. Procoagulant and proinflammatory intramyocardial (micro)vasculature plays an important role in acute myocardial infarction (AMI). Dysfunction of the intramyocardial microvasculature, specifically the endothelium, following AMI, is suggested to relate to left ventricle remodeling, contractility, and cardiac events, such as reinfarction and death. Also, hindered perfusion of the ischemic myocardium after restoration of coronary flow, known as no-reflow or coronary microvascular obstruction (MVO), might point to dysfunctional coronary microvasculature and is associated with larger infarct size and poor clinical outcome. The endothelial DPP4 expression and activity were studied in human myocardial infarction in relation to a prothrombogenic endothelial phenotype.

**Figure 1:**
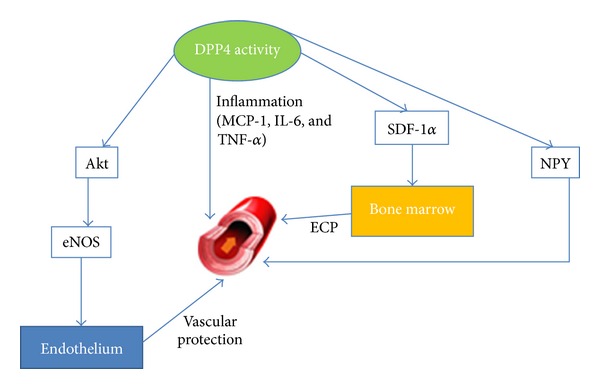
Effect of DPP4 on cardiovascular districts.

Another known DPP4 substrate is fibrin and via cleaving N-terminal Gly-Pro from the fibrin a-chain, DPP4 can inhibit fibrin polymerization and clot formation [28]. As such DPP4 may behave as an immobilized anticoagulant on (microvascular) endothelium, DPP4 expression and activity were found on the endothelium of intramyocardial blood vessels in autopsied control hearts. Within the infarction area of AMI patients, this DPP4 expression and activity were significantly decreased, corresponding to an increase in Tissue Factor expression. In primary human umbilical vein endothelial cells (HUVECs), DPP4 expression was strongly decreased after metabolic inhibition, also inducing Tissue Factor upregulation. Aggregation of platelets to endothelial cells is a sign of thrombogenicity. Under these conditions it was tested whether inhibition of DPP4 activity by diprotin A in HUVECs leads to increased adherence of nonstimulated platelets under flow conditions. The inhibition of DPP4 activity with diprotin A enhanced the amount of Tissue Factor encountered and induced the adherence of platelets under flow conditions. Ischemia induces loss of coronary microvascular endothelial DPP4 expression and increased Tissue Factor expression in AMI as well as in vitro in HUVECs. Therefore, the reduced presence/activity of DPP4 and increased presence of Tissue Factor in the post-MI intramyocardial microvasculature suggest a shift towards a prothrombogenic status of the endothelium and an increased risk of intramyocardial thrombosis. The loss of DPP4 activity affects the antithrombogenic nature of the endothelium [29]. These effects are summarized in Figure 1.

## 4. Conclusions

The study of DPP4 enzymatic activity has been focused in recent years on metabolic effects linked to degradation of GLP-1. Actually DPP4 is known to be present in many cells and tissues and its effects go beyond purely metabolic aspects. Almost always the inhibition activity of DPP4 is associated with improved cardiovascular profile, but it has shown to possess antithrombotic properties and the different actions could be connected with a site and/or species specificity of DPP4. Certainly, DPP4 seems to exert many functions both directly and indirectly on cardiovascular districts, opening new possibilities of prevention and treatment of cardiovascular complications not only in patients with diabetes mellitus.
